# Implementation of the recommendations of the II Brazilian Consensus On Gastric Cancer in clinical practice: a multicenter study of the Brazilian Gastric Cancer Association

**DOI:** 10.1590/0102-67202025000027e1896

**Published:** 2025-09-01

**Authors:** Marcus Fernando Kodama Pertille RAMOS, Marina Alessandra PEREIRA, Alexandre Farias de ALBUQUERQUE, Eduardo Freitas VIANA, Wilson Luiz COSTA, Soraya Rodrigues de Almeida SANCHES, Andre Maciel SILVA, Ulysses RIBEIRO, Andrea Pedrosa Ribeiro Alves OLIVEIRA, Felipe Carvalho VICTER, Giovanni Zenedin TARGA, Paulo Pimentel ASSUMPÇÃO, Antonio Carlos WESTON, João Paulo RIBEIRO, Luis Fernando MOREIRA, Fatima MRUE, Luiz Roberto LOPES, Paulo KASSAB, João Odilo Gonçalves PINTO, Leandro Cardoso BARCHI, Nora Manoukian FORONES

**Affiliations:** 1 Universidade de São Paulo, Faculty of Medicine, Hospital das Clinicas, Cancer Institute, Department of Gastroenterology – São Paulo (SP), Brazil.; 2 Hospital Aristides Maltez, Bahian League Against Cancer – Salvador (BA), Brazil.; 3 Universidade Federal da Bahia, Hospital Universitário Professor Edgard Santos – Salvador (BA), Brazil.; 4 AC Camargo Cancer Center – São Paulo (SP), Brazil.; 5 Universidade Federal de Minas Gerais, Hospital Universitário – Belo Horizonte (MG), Brazil.; 6 National Cancer Institute – Rio de Janeiro (RJ), Brazil.; 7 Universidade de Brasília, Hospital Universitário de Brasília – Brasília (DF), Brazil.; 8 Universidade Federal do Rio de Janeiro, Hospital Universitário Clementino Fraga Filho – Rio de Janeiro (RJ), Brazil.; 9 Hospital Erasto Gaertner – Curitiba (PR), Brazil.; 10 Universidade Federal do Pará, Hospital Universitário João de Barros Barreto – Belém (PA), Brazil.; 11 Santa Casa de Misericórdia de Porto Alegre – Porto Alegre (RS), Brazil.; 12 Universidade Federal de Pernambuco, Hospital Universitário – Recife (PE), Brazil.; 13 Hospital Universitário de Porto Alegre – Porto Alegre (RS), Brazil.; 14 Universidade Federal de Goias, Hospital Universitário – Goiânia (GO), Brazil.; 15 Universidade Estadual de Campinas, Hospital Universitário – Campinas (SP), Brazil.; 16 Santa Casa de Misericórdia de São Paulo – São Paulo (SP), Brazil.; 17 Universidade Federal do Ceará, Hospital Universitário Walter Cantídio – Fortaleza (CE), Brazil.; 18 Brazilian Gastric Cancer Association – São Paulo (SP), Brazil.

**Keywords:** Gastric Cancer, Consensus, Guideline as Topic, Gastrectomy, Lymphadenectomy, Câncer Gástrico, Consenso, Guias como Assunto, Gastrectomia, Linfadenectomia

## Abstract

**Background:**

The management of gastric cancer has become increasingly complex, highlighting the importance of clinical guidelines to ensure standardized care. The Second Brazilian Consensus on Gastric Cancer was developed to guide clinical practice across the country.

**Aims:**

The aim of this study was to evaluate the degree of implementation of the 2nd Brazilian Consensus recommendations in cancer reference centers in Brazil.

**Methods:**

This multicenter study involved 18 cancer centers that prospectively collected data over a one-year period. Notably, 21 key statements from the Consensus were assessed. Adherence was defined as following the recommendation in more than 80% of applicable cases.

**Results:**

Of the 21 statements, 11 (52.4%) met the predefined adherence threshold. The selective use of endoscopic ultrasound and PET-CT was consistent with the recommendations. However, diagnostic laparoscopy was underutilized, performed in only 24.7% of patients. Preoperative nutritional therapy, another key recommendation, was provided in just 42% of cases. D2 lymphadenectomy was performed in 79.8% of surgeries, but only 63.3% of specimens included ≥25 lymph nodes, the recommended minimum for adequate staging. Minimally invasive surgery (MIS) was performed in approximately 25% of early distal tumors but was rarely used in advanced proximal tumors. Despite not being recommended for early stage tumors, omentectomy and bursectomy were still performed in a significant number of T1/T2 cases. Preoperative chemotherapy was used in 35.4% of distal tumors ≥IB and 54.3% of proximal tumors, showing partial adherence to this recommendation.

**Conclusions:**

Just over half of the II Brazilian Consensus recommendations were implemented in routine practice. There was strong adherence to D2 lymphadenectomy and MIS for early distal tumors. However, there is still room for improvement in areas such as diagnostic laparoscopy, nutritional support, adequate lymph node retrieval, and using more neoadjuvant chemotherapy to enhance care and follow national guidelines.

## ARTICLE HIGHLIGHTS

- Gastric cancer (GC) remains a major global health problem. Despite a decline in its incidence, GC is still the third most lethal cancer worldwide.
- Multimodal treatment approaches are employed, including chemotherapy (CMT), radiotherapy (RDT), surgery, expanded criteria for endoscopic resection, and increased use of minimally invasive surgery.
- The development of clinical guidelines and consensus recommendations to update and guide healthcare professionals involved in GC treatment has gained increasing prominence.
- Preoperative nutritional therapy, indication of D2 lymphadenectomy, and the use of minimally invasive surgery for distal EGC, was notably strong.
- Greater attention is warranted regarding the broader implementation of diagnostic laparoscopy and ensuring the retrieval of an adequate number of lymph nodes during D2 lymphadenectomy to optimize staging and outcomes.

## CENTRAL MESSAGE

The development of consensus statements and clinical guidelines supports decision-making in clinical practice. However, recommendations formulated by experts may not always reflect real-world clinical practice. In this study, 21 key statements from the 2nd Brazilian Consensus on Gastric Cancer were evaluated across multiple cancer reference centers. It was found that, in 10 of these statements, current clinical practice diverged from the consensus recommendations.

## PERSPECTIVES

Some consensus statements may be revised in future editions to better reflect the realities of clinical practice in the national context. To enhance adherence to the recommendations, broad dissemination of the study results is essential, alongside the implementation of educational initiatives and institutional policies aimed at promoting guideline compliance. These measures may contribute to closing the gap between consensus recommendations and everyday clinical practice, ultimately improving patient outcomes.

## VISUAL ABSTRACT

**Figure:**
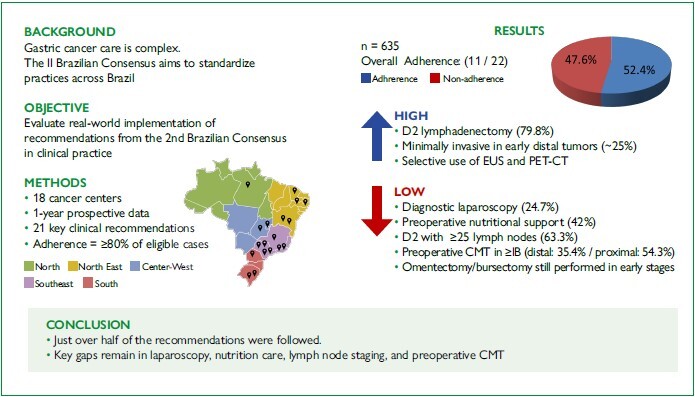

## RESUMO

**Racional:**

O manejo do câncer gástrico tornou-se cada vez mais complexo, ressaltando a importância de diretrizes clínicas e consensos especializados para padronizar o cuidado. O 2º Consenso Brasileiro sobre Câncer Gástrico foi elaborado com o objetivo de orientar a prática clínica em todo o país.

**Objetivos:**

Avaliar o grau de implementação das recomendações do 2o Consenso Brasileiro sobre Câncer Gástrico em centros de referência oncológica no Brasil.

**Métodos:**

Estudo multicêntrico com participação de 18 centros especializados no tratamento do câncer, que coletaram dados prospectivamente ao longo de um ano. Foram analisadas 21 declarações-chave do Consenso. Considerou-se aderência quando a declaração foi seguida em mais de 80% dos casos aplicáveis.

**Resultados:**

Das 21 declarações avaliadas, 11 (52,4%) atingiram o critério de adesão. O uso seletivo da ultrassonografia endoscópica e do PET-CT esteve de acordo com o Consenso. No entanto, a laparoscopia diagnóstica foi subutilizada, sendo realizada em apenas 24,7% dos pacientes. A terapia nutricional pré-operatória foi oferecida em 42% dos casos. A linfadenectomia D2 foi realizada em 79,8% das cirurgias, mas apenas 63,3% das peças continham ≥25 linfonodos, número recomendado para estadiamento adequado. A cirurgia minimamente invasiva foi utilizada em cerca de 25% dos tumores distais precoces, sendo rara nos tumores proximais avançados. Apesar de não recomendadas para tumores iniciais, omentectomia e bursectomia ainda foram realizadas em número significativo de casos T1/T2. A quimioterapia pré-operatória foi utilizada em 35,4% dos tumores distais estádio ≥IB e em 54,3% dos proximais, evidenciando adesão parcial.

**Conclusões:**

Pouco mais da metade das recomendações do 2o Consenso Brasileiro foram implementadas na prática. Houve boa adesão à linfadenectomia D2 e à cirurgia minimamente invasiva para tumores distais precoces. No entanto, a laparoscopia diagnóstica, o suporte nutricional, a linfadenectomia adequada e o uso mais amplo da quimioterapia neoadjuvante ainda exigem melhorias.

## INTRODUCTION

 Gastric cancer (GC) remains a major global health problem. Despite a decline in its incidence, GC is still the third most lethal cancer worldwide^38^. Fortunately, recent advances in treatment have contributed to improved survival for diagnosed patients. Multimodal treatment approaches, including chemotherapy (CMT), radiotherapy (RDT), surgery, expanded criteria for endoscopic resection, and increased use of minimally invasive surgery (MIS), are some examples that have advanced GC management^5,21^. 

 In this context, the development of clinical guidelines and consensus recommendations to update and guide healthcare professionals involved in GC treatment has gained increasing prominence. The Japanese Gastric Cancer Association (JGCA) published its first treatment guideline in 2001, with the sixth and most recent edition released in English in 2024^21,28^. In addition to the JGCA guidelines, other regional guidelines have been published, notably from Europe, North America, and South Korea^3,23,26^. 

 Founded in 1999, the Brazilian Gastric Cancer Association (ABCG) unites specialists involved in GC treatment in Brazil, with a focus on continuing education and professional training. The first Brazilian Consensus on Gastric Cancer was published in 2013^41^, followed by an updated consensus in 2020 that incorporated new therapeutic options for GC^5,6^. 

 The second consensus included 67 statements covering diagnosis, staging, and treatment. However, some statements revealed disagreement among the specialist panel and appeared challenging to implement in practice. Therefore, the present study aims to evaluate the implementation of the 2^nd^ Brazilian Consensus recommendations in clinical practice across Brazil. 

## METHODS

 This study is part of the ABCG (Brazilian Association of Gastric Cancer) project, which evaluated the overview of GC treatment in Brazil using a prospectively collected database. The research methodology has been previously published^31^. Briefly, this is a multicenter study involving 18 centers with recognized expertise in GC treatment. At least one center from each Brazilian region participated, ensuring geographic representation that mirrors the population distribution. 

 The project included patients diagnosed with gastric adenocarcinoma who underwent any surgical procedure for GC treatment between June 2022 and June 2023. Patients with other histological tumor types (such as GIST, neuroendocrine tumors, or lymphoma) and those undergoing surgery solely for complications related to metastatic disease were excluded. 

 During the study design, 21 statements from the 2nd Brazilian Consensus were selected for evaluation^5^. These statements were chosen based on reported difficulties in adoption within Brazilian practice or because they related to new therapeutic modalities assessable through the collected variables. The consensus methodology has been described elsewhere^5^. In brief, consensus was considered achieved if more than 80% of responses agreed fully or partially with the statements. Similarly, in this study, adherence to the consensus was defined as following the recommendation in more than 80% of eligible cases. 

 The evaluation covered staging and preoperative care characteristics such as endoscopic ultrasound (EUS), positron emission tomography (PET-CT) scan, diagnostic laparoscopy, and nutritional support. Surgical aspects assessed included the type of lymphadenectomy, number of lymph nodes retrieved, extent of resection, use of minimally invasive surgery (MIS; laparoscopic and robotic), omentectomy, and bursectomy. 

 Technical details such as abdominal drain usage, duodenal stump closure technique, reconstruction type, and esophago-jejunal anastomosis method were also recorded. Additionally, adjuvant radiotherapy (RDT), adjuvant chemotherapy (CMT), and perioperative CMT according to tumor location were evaluated. Surgical and multimodal treatment assessments were limited to patients undergoing gastrectomy with curative intent. 

 Data were maintained independently by each center in a database built on the REDCap web platform. The study was approved by the Ethics Committees of all participating centers and registered on Plataforma Brasil (CAAE: 41844820.5.1001.0068). The Hospital das Clínicas of the University of São Paulo Medical School (CCEP 1833/20) served as the main coordinating center. Patient informed consent was waived. 

 Continuous variables are presented as mean ± standard deviation (SD) or median with interquartile range (IQR), and categorical variables as counts and percentages. Percentages were calculated excluding missing data to a total of 100%. The chi-square test was used to compare categorical variables, and the t-test for continuous variables. Statistical analyses were conducted using SPSS version 20 (IBM, Chicago, IL). 

## RESULTS

 A total of 635 patients with GC were enrolled in the study. The mean age was 62 years, and 58.3% of the patients were male. Regarding treatment, 464 patients (73.1%) underwent gastrectomy (either total or subtotal) with curative intent. Out of the 21 evaluated consensus statements, 5 reached consensus and were implemented in clinical practice in more than 80% of eligible patients (Table 1). Another six statements did not reach consensus and were also followed in less than 80% of cases. Finally, there was disagreement between the consensus recommendations and real-world practice in 10 statements. 

**Table 1:** Demographic and surgical characteristics of patients undergoing orthotopic liver transplantation.

Number	Statement		II Brazilian Consensus (%)	Project overview ABCG (%)	Compliance with consensus
	Diagnosis statements				
3		Ultrasound upper endoscopy is not indicated when there are clear endoscopic signs that the cancer is invasive. It should be used when there is any doubt about the early aspect of GC. It allows for evaluation of the degree of tumor invasion in the gastric wall and the presence of suspicious lymph nodes for metastases.	96	96.6	ü
5		Positron emission tomography (PET-CT) and nuclear magnetic resonance (MRI) should be used only in selected cases.	100	97.6	ü
7		Staging laparoscopy should be performed in cases where there is uncertainty in computed tomography regarding the presence of peritoneal.	98	24.7	û
	Treatment statements				
12		Patients who have had weight loss greater than 10% of their usual weight in the past 6 months should receive some form of nutritional therapy before starting any treatment.	100	42	û
17		In stage IB-III tumors (T2-4 any N), D2 lymph node dissection is indicated.	98	79.6	û
18		The UICC/AJCC recommends a minimum of 15 harvested lymph nodes to allow correct staging.	92	89.1	ü
19		D2 lymphadenectomy recommends at least 25 harvested lymph nodes.	76	63.3	ü
37		Laparoscopic subtotal gastrectomy can be performed in distal third early GC.	98	25.3	û
38		Laparoscopic subtotal gastrectomy can be performed in distal third advanced gastric cancer.	92	9.2	û
39		Laparoscopic total gastrectomy can be performed in upper third early gastric cancer.	90	24.2	û
40		Laparoscopic total gastrectomy can be performed in upper third advanced GC.	76	7.1	ü
34		Partial omentectomy (up to 3–5 cm from the gastroepiploic arcade) can be performed on T1/T2 tumors, and total omentectomy must be performed on T3/T4 tumors.	78	66	ü
35		Bursectomy should be performed only on T4 tumors arising from the posterior gastric wall.	80	40.2	û
47		Routine abdominal drain(s) are recommended for all gastric resections.	70	65.1	ü
48		The duodenal stump should preferably be closed using mechanical suture.	84	98.7	ü
50		In subtotal and total gastrectomies, digestive transit should preferably be reconstructed by Roux-en-Y derivation.	96	91.1	ü
51		Gastrojejunostomy and esophagojejunostomy should preferably be performed with mechanical suture.	70	60	ü
	Chemoradiotherapy statements				
53		Perioperative chemotherapy (before and after surgery) is indicated for stage ≥IB resectable tumors of the distal third.	82	35.2	û
54		Perioperative chemotherapy (before and after surgery) is indicated for stage ≥IB resectable tumors of the middle and proximal third.	78	54.2	ü
55		Stage ≥IB patients who underwent surgery without perioperative chemotherapy (upfront surgery) have an indication for adjuvant chemotherapy.	80	41.3	û
56		Adjuvant radiotherapy is recommended in cases with an indication for adjuvant chemotherapy and who did not have an adequate lymph node dissection during surgery.	82	2.7	û

ABCG: Brazilian Gastric Cancer Association; GC: Gastric cancer; UICC: Union Internationale Contre le Cancer; AJCC: American Joint Committee on Cancer.

### Diagnosis and preoperative treatment statements

**Figure 1:**
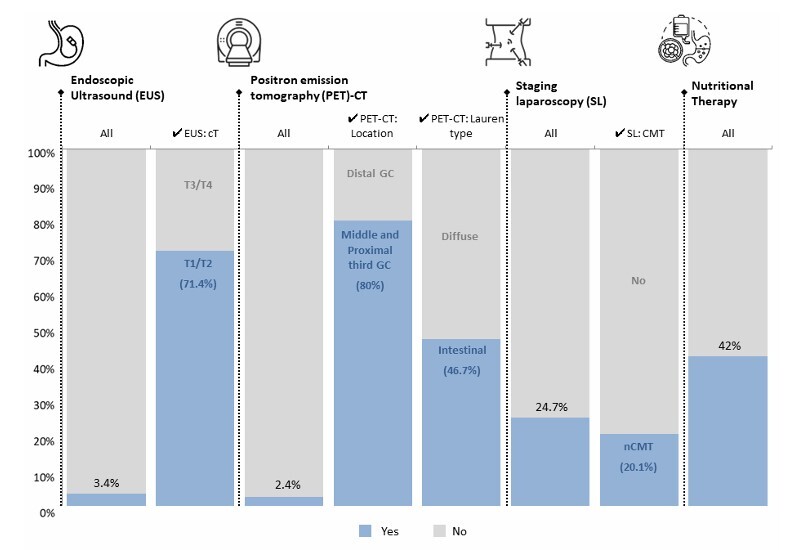
Frequency of endoscopic ultrasound, positron emission tomography-computed tomography, staging laparoscopy, and preoperative nutritional therapy. EUS: endoscopic ultrasound; PET-CT: positron emission tomography-computed tomography; SL: staging laparoscopy; CMT: chemotherapy.

 In total, four statements related to staging examinations and preoperative care were evaluated (Figure 1). Regarding EUS, only 3.4% of patients underwent the procedure, of whom 71.4% had clinical T1 or T2 tumors. A PET scan was performed in 2.4% of patients; among these, 80% had tumors located in the proximal or middle third of the stomach and 46.7% were of the intestinal histological subtype. Diagnostic laparoscopy was conducted in 24.7% of cases overall. When considering only patients with an indication for preoperative chemotherapy (n=433; cT2–cT4; and/or cN+), 20.1% underwent diagnostic laparoscopy. Preoperative nutritional therapy was administered to 42% of patients. Among these, the mean hemoglobin level was 11.5 g/dL (standard deviation — SD±2.6), and 46.9% had low hemoglobin (Hb) and/or albumin (Alb) levels (Hb < 11 g/dL and Alb <3.5 g/dL), compared to 30% in patients who did not receive nutritional therapy (p=0.001, p<0.05). Additionally, patients receiving nutritional therapy had a lower body mass index (BMI) compared to those who did not (23.4±5.2 vs. 24.7±5.4 kg/m^2^, p=0.003, p<0.05). 

### Lymph node dissection and extension of lymphadenectomy

**Figure 2:**
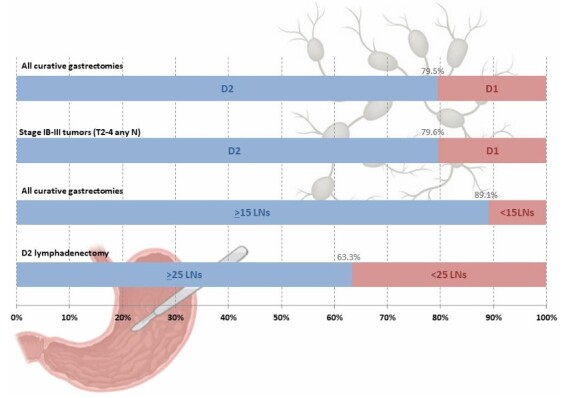
Characteristics of lymphadenectomy performed, including the frequency of D2 dissection and adequacy of lymph node retrieval. LN: lymph nodes.

 The Consensus panel of specialists recommended D2 lymphadenectomy as the standard surgical dissection for stage IB-III gastric cancer. In the present study, 20.4% of patients meeting the indication criteria did not undergo D2 lymphadenectomy (Figure 2). Among these patients, 47.3% were over 70 years old, 33.3% had an American Society of Anesthesiology (ASA) score of III or IV, and 16.5% had an ECOG (East ern Cooperative Oncology Group) performance status of 2–3, compared to 20.8%, 19.9%, and 9.2%, respectively, in those who underwent D2 dissection (all p<0.05). Adequate lymph node staging, defined by the retrieval of at least 15 lymph nodes as recommended by consensus statement number 18, was achieved in 81.9% of cases. However, the more stringent criterion of retrieving over 25 lymph nodes in D2 dissections was met in only 63.3% of cases. 

### Surgical aspects and techniques

 Among all curative-intent gastrectomies, 55% were subtotal and 45% were total resections. MIS was performed in 15.8% of cases (n=63), of which 60.3% (38 cases) were subtotal and 39.7% (25 cases) were total gastrectomies. 

**Figure 3:**
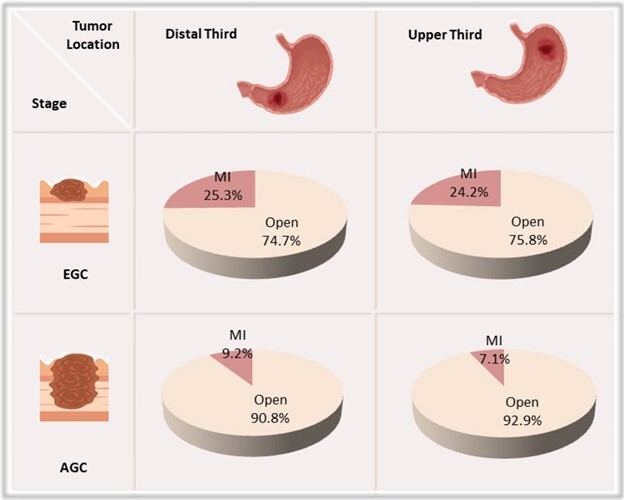
Frequency of minimally invasive surgery according to tumor location and stage in gastric cancer patients. MI: minimally invasive; EGC: early gastric cancer; AGC: advanced gastric cancer.

 The 2nd Consensus reported 98% agreement that laparoscopic gastrectomy is appropriate for distal early gastric cancer (EGC) and 92% agreement for distal advanced GC (AGC)^5^. In this study, of the subtotal gastrectomies performed via MI access, 63.2% were distal EGC and 36.8% distal AGC. Overall, 25.4% of subtotal EGC cases and 9.2% of subtotal AGC cases were operated on using a minimally invasive approach (Figure 3). 

 For proximal tumors requiring total gastrectomy, there was 90% consensus supporting laparoscopic access for EGC and 76% for AGC. Among the 25 total gastrectomies performed minimally invasively, 60% were proximal EGC and 40% were advanced tumors. Considering all total gastrectomies (both MI and open), 24.2% of EGC and 7.1% of AGC cases were performed via MIS. 

**Figure 4:**
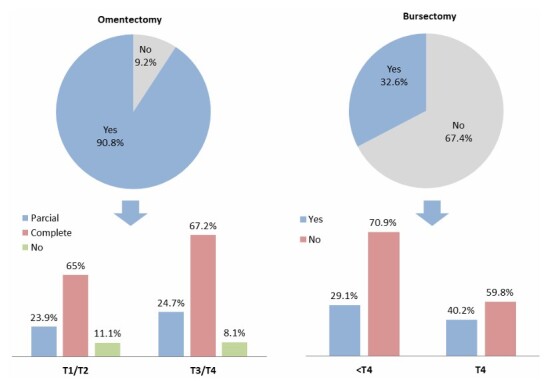
Frequency of omentectomy and bursectomy procedures according to tumor stage.

 There was no consensus among the panel of specialists regarding the indication for partial omentectomy in T1 and T2 GC, nor for complete omentectomy in T3 and T4 tumors (Figure 4). In this study, total omentectomy was performed in 65% of patients with T1/T2 tumors and 67.2% of those with T3/T4 GC. Statement number 35 reached 80% consensus, recommending bursectomy only for T4 tumors of the posterior gastric wall. However, bursectomy was performed in 59.8% of all T4 cases in the present study, and notably, 29.1% of patients with T1, T2, or T3 tumors also underwent bursectomy. 

**Figure 5:**
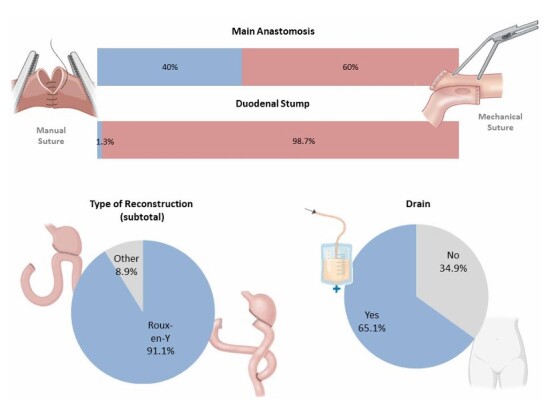
Characteristics of surgical techniques: main anastomosis method, duodenal stump closure, reconstruction type, and use of abdominal drains.

 There was no consensus (70% agreement) regarding the routine use of abdominal drainage, which was performed in 65.1% of patients (Figure 5). The Consensus recommended mechanical stapling for duodenal stump closure with 84% agreement, and this technique was used in 98.7% of cases. Roux-en-Y reconstruction was endorsed by 96% of panelists and was performed in 91.1% of patients undergoing sub total gastrectomy. However, there was no consensus (70%) on the preferred method for the main anastomosis, and we found that 60% of the anastomosis was performed with mechanical suture. 

### Multimodal treatment

**Figure 6:**
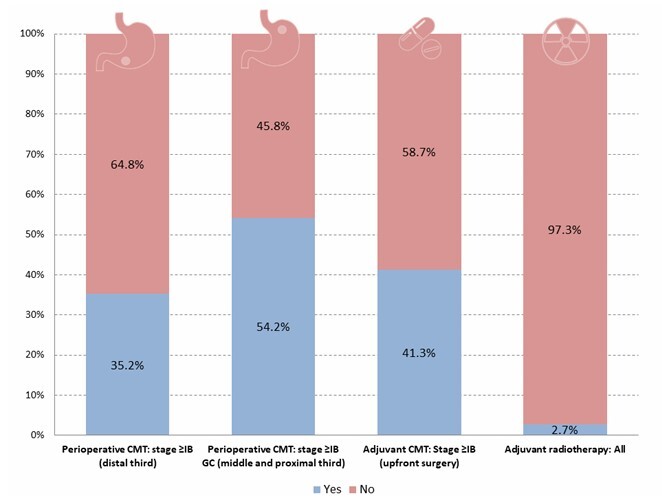
Frequency of multimodal treatment approaches, including perioperative chemotherapy, upfront surgery, adjuvant chemotherapy, and adjuvant radiotherapy. CMT: chemotherapy.

 The indication of perioperative CMT for distal tumors staged >IB was agreed upon by 82% of the panelists; however, 64.8% of these patients underwent upfront surgery instead. For proximal tumors, 78% of the consensus recommended perioperative CMT, but upfront surgery was performed in 45.8% of cases (Figure 6). Consequently, preoperative CMT was administered to 35.2% of patients with distal GC and 54.2% with proximal GC. 

 Among patients who did not receive preoperative CMT, there was 80% consensus supporting adjuvant chemotherapy for pathological stage ≥IB. In practice, only 41.3% of patients with such indications received adjuvant CMT. Additionally, there was 82% consensus recommending adjuvant RDT when lymphadenectomy was inadequate. Despite this, RDT was indicated in just 2.7% of cases, and none of these had fewer than 15 lymph nodes dissected or an R1 resection. 

## DISCUSSION

 This study evaluated the implementation of 21 statements from the 2nd Brazilian Consensus on Gastric Cancer in routine clinical practice across Brazil^5^. Among the 15 statements that reached consensus agreement, only 5 were fully applied in practice. The remaining 10 consensus-agreed statements were not consistently followed in daily clinical care. Additionally, of the 6 statements that did not reach consensus, none were effectively adopted in practice (adoption in fewer than 80% of cases). Overall, 11 out of the 21 evaluated parameters aligned with the 2nd Consensus recommendations, resulting in a theory-to-practice implementation rate of 52.4%^5^. Although this rate may appear low, some consensus statements were formulated in ways that are challenging to implement in the Brazilian clinical setting, as will be discussed below. 

 There was a low rate of EUS and PET utilization for staging. According to current consensus guidelines, the primary indications for EUS include assessing the depth of tumor invasion within the gastric wall, particularly to determine eligibility for endoscopic resection in EGC, and evaluating lymph node involvement to guide the indication for preoperative CMT. In our study, most patients who underwent EUS had T1/T2 GC, indicating that the evaluation for potential en doscopic resection remains the main reason for its use. However, patients who ultimately underwent endoscopic resection were not included in the present analysis. Although upper digestive endoscopy can accurately assess submucosal invasion, EUS is still frequently performed as part of the staging workup when endoscopic resection is being considered^8,39^. Therefore, it is possible that the actual frequency of EUS use is higher than reported, considering the exclusion of patients who underwent endoscopic resection^18,37^. 

 The use of PET scans and diagnostic laparoscopy as complementary tools for staging has yielded differing results. According to current consensus guidelines, PET scans should be reserved for selected cases, and indeed, its use was infrequent in our cohort. The limited indication is likely due to the known low sensitivity of PET in detecting GC, particularly in diffuse-type tumors, as well as its high cost^16^. In contrast, diagnostic laparoscopy was performed in approximately 25% of patients. Despite the challenges related to operating room availability, commonly encountered in the public healthcare system, this rate is considered reasonable. Literature reports indicate that laparoscopy can lead to a change in treatment strategy in up to 30% of cases. Furthermore, its role in enabling patient inclusion in clinical trials involving intraperitoneal therapies underscores the importance of expanding its use in staging protocols^14,35^. 

 The indication for nutritional therapy in patients with weight loss greater than 10% over 6 months reached 100% agreement in the Consensus, reflecting the well-established benefits of nutritional support in the perioperative management of surgical patients. GC is a disease strongly associated with malnutrition, driven both by cancer cachexia, mediated by systemic inflammation, and by reduced oral intake due to tumor-related symptoms^15^. Globally, various initiatives have promoted the implementation of nutritional therapy, and in Brazil, the ACERTO (Acceleration of Total Post-Operative Recovery) Project stands out as a key program. Through publications, seminars, and educational activities, this initiative has contributed significantly to the dissemination of best practices in perioperative care. The unanimous agreement on the indication of nutritional therapy, along with its effective adoption in clinical practice, highlights the project’s impact^1,2^. In our study, 42% of patients received nutritional therapy. Although the route of administration (oral, enteral, or parenteral) was not specified, this rate may appear low at first glance. However, it is important to note that the presence or extent of weight loss was not evaluated in the current analysis. Thus, it is plausible that many patients who did not receive nutritional therapy simply did not meet the clinical criteria for its initiation. Supporting this hypothesis, patients who did receive nutritional therapy had significantly lower levels of hemoglobin, albumin, and BMI; parameters commonly associated with poor nutritional status. 

 The indication for D2 lymphadenectomy reached a 98% consensus among experts; however, in clinical practice, only 80% of patients underwent the procedure. Historically, the role of D2 lymphadenectomy in Western countries was debated due to its greater technical complexity and associated morbidity and mortality^17^. Fortunately, in Brazil, the indication for D2 lymphadenectomy is now well-established. Its omission in a subset of patients in this study is likely attributable to poor clinical status, particularly in older individuals with higher ASA and ECOG scores, limiting their ability to tolerate more extensive surgery^33^. A noteworthy and somewhat contradictory finding of the study was that 35% of surgeries classified as D2 lymphadenectomy involved the dissection of fewer than 25 lymph nodes. Notably, two main hypotheses may explain this discrepancy. The first is the actual non-performance of a true D2 lymphadenectomy, possibly due to technical limitations or deviations during surgery^12^. The second, and perhaps more prevalent, reason could be the inadequate handling of the surgical specimen, particularly with respect to lymph node retrieval. Best practices recommend that the surgeon either dissect the lymph nodes or at least separate nodal stations before sending the specimen to pathology. Unfortunately, this crucial step is sometimes overlooked, especially when communication between the surgical and pathology teams is limited. The use of lymph node-revealing solutions may aid in recovering a greater number of nodes, but ultimately, optimal staging relies on close collaboration between the surgeon and pathologist^13^. This finding underscores the need for increased attention to the quality of lymphadenectomy and lymph node retrieval, ensuring that both surgical execution and pathological assessment align with established oncological standards. 

 The introduction of laparoscopy for the treatment of GC gained significant momentum in Asia, where its adoption followed a stepwise approach, initially for early distal tumors, then for advanced cases, and finally for proximal tumors^19,24,40^. This progression was guided by oncological caution, particularly the concern that MI surgery might compromise the quality of lymphadenectomy in AGC. Distal tumors were prioritized not only due to their more favorable prognosis but also because total gastrectomy requires the dissection of a greater number of lymph node stations and involves technically demanding esophagojejunal anastomoses. Large randomized controlled trials (RCTs) from Asia followed this same trajectory, and in our study, we observed a similar pattern: mini mally invasive gastrectomy was more frequently performed in distal EGC and less frequently in proximal AGCs. This finding reflects a good alignment of clinical practice in Brazil with international standards and literature^22,30^. As a benchmark, over 70% of patients in South Korea currently undergo MIS for GC^20^. Achieving such high rates in Brazil is unlikely in the near term, given economic constraints and local characteristics such as a higher prevalence of AGC and a greater proportion of patients with elevated BMI. Nonetheless, the growing implementation of MIS in our context is encouraging and supports further expansion of its use in Brazil. 

 The role of omentectomy and bursectomy in the surgical treatment of GC has diminished in recent years^7,25^. Updates in the latest Japanese guidelines have limited the indication for omentectomy to tumors classified as T3 or deeper, while the routine use of bursectomy has been abandoned due to a lack of demonstrated oncological benefit. Despite these changes, the proportion of patients undergoing both procedures in Brazil remains relatively high. Although these interventions were more commonly performed in patients with advanced tumors, as per guideline recommendations, our findings may also reflect a lag in the adoption of updated surgical practices. This suggests the need for continued efforts to align clinical practice with current evidence and international standards. 

 In the evaluation of technical aspects, our findings demonstrated a clear lack of consensus regarding the use of surgical drains and the preferred technique for esophagojejunostomy. Although enhanced recovery protocols, such as ERAS (enhanced recovery after surgery), discourage the routine use of drains, this practice remains common in clinical settings^34^. It is important to highlight that limited access to interventional radiology in many Brazilian institutions contributes to surgeons’ reluctance to omit drains, due to concerns about managing potential postoperative collections or guiding fistula treatment should complications arise. Conversely, recent advances in endoscopic management, particularly intraluminal vacuum therapies, have significantly reduced the reliance on surgical drains for the treatment of anastomotic fistulas^11,36^. In this context, the future expansion of interventional radiology services and the growing expertise in advanced endoscopic techniques may contribute to a gradual reduction in drain usage in routine surgical practice. 

 While the technique for esophagojejunostomy remains non-standardized, nearly all patients in our study underwent mechanical stapling for duodenal stump closure. Duodenal stump fistula continues to be one of the most feared postoperative complications, particularly because it lacks a viable option for intraluminal endoscopic treatment. To date, there is no definitive evidence linking the occurrence of duodenal stump fistula to the choice between manual suturing and stapling techniques. The primary risk factor appears to be the presence of distal tumors, which necessitate a more distal transection of the duodenum and may increase tension at the stump^32^. 

 Finally, the findings related to multimodal therapy helped clarify a previously questionable result from the 2nd Consensus^5^. Specifically, the Consensus showed greater agreement on the indication of preoperative CMT for distal tumors staged as ≥IB, compared to proximal tumors^9^. This result was unexpected, given that one of the main criticisms of early studies on preoperative CMT was the predominance of proximal and esophagogastric junction tumors over distal tumors in their patient populations^4,10,41^. In our study, clinical practice aligned more closely with the available evidence, as preoperative CMT was more frequently indicated for proximal GCs than for distal ones, mirroring the patient profile of the major trials that evaluated this treatment approach. An additional factor contributing to the lower use of preoperative CMT in distal tumors may be the higher incidence of obstructive lesions in this location, which often compromises the patient’s ability to tolerate systemic therapy prior to surgery. Moreover, the use of RDT in the adjuvant setting for gastric cancer has declined significantly. In our cohort, less than 3% of patients received radiotherapy. Notably, none of these patients had fewer than 15 dissected lymph nodes and a positive resection margin, criteria that traditionally justified adjuvant radiotherapy in older treatment paradigms^27,29^. 

## CONCLUSIONS

 There was agreement between consensus statements and clinical practice in approximately half of the evaluated recommendations. Although this proportion may appear modest, the alignment of key procedures, such as preoperative nutritional therapy, indication of D2 lymphadenectomy, and the use of MIS for distal EGC, was notably strong. Nonetheless, greater attention is warranted regarding the broader implementation of diagnostic laparoscopy and ensuring the retrieval of an adequate number of lymph nodes during D2 lymphadenectomy to optimize staging and outcomes. 

## Data Availability

The datasets generated and/or analyzed during the currentstudy are available from the corresponding author upon reasonable request.
